# The particle-gravity frontier

**DOI:** 10.1098/rsta.2023.0093

**Published:** 2024-02-05

**Authors:** Steven D. Bass, Julia Harz, Lavinia Heisenberg

**Affiliations:** ^1^ Kitzbühel Centre for Physics, Kitzbühel, Austria; ^2^ Marian Smoluchowski Institute of Physics, Jagiellonian University, Kraków, Poland; ^3^ PRISMA^+^ Cluster of Excellence & Mainz Institute for Theoretical Physics, Johannes Gutenberg-Universität Mainz, 55099 Mainz, Germany; ^4^ Institute for Theoretical Physics, University of Heidelberg, Heidelberg, Germany

This theme issue concerns exciting new developments in our quest to understand the deep structure of the Universe: the interface of the particle physics and gravity frontiers. The particle physics Standard Model provides an excellent description of the subatomic world measured in present experiments. Likewise, General Relativity describes our present measurements of gravitation. Yet we know that there must be new physics waiting to be discovered and understood. Open puzzles include the nature of neutrinos (whether they are Dirac or Majorana particles) and the generation of their masses, the mechanism behind the matter-antimatter asymmetry in the Universe, why there are three families of fermions in particle physics, and the nature of dark matter and dark energy.

The challenge to understand the deep structure of matter requires close interaction between experiments and theory. How high in energy and precision can we push the Standard Model before new physics will show up? Might we see variations from the predictions of General Relativity in future measurements of gravitation? The search for cracks in the present theories involves a combination of high-energy collider and low-energy precision experiments, astroparticle and gravitational wave physics, and cosmology surveys. These are driven by the combined creativity of designing new experiments and fresh theoretical thinking.

The Higgs boson discovered at CERN behaves very Standard Model like and completes the spectrum of the Standard Model [1,2]. In the Standard Model, the Higgs mechanism is responsible for the masses of elementary particles with the masses of the weak W and Z gauge bosons as well as the quarks and charged leptons related to their coupling to the Higgs boson. In total, 15 of the 18 parameters of the Standard Model are connected to the Higgs sector. In addition, neutrino oscillations suggest that neutrinos come with tiny masses, with the precise values awaiting measurement and accompanying theoretical understanding [3]. Might neutrinos be their own antiparticles, so-called Majorana fermions? If so, will we be able to discover lepton-number violation at neutrinoless double beta decay experiments, or at the LHC or in meson decays?

To explain the matter-antimatter asymmetry, the three renowned Sakharov conditions have to be fulfilled: (i) B-L violation, (ii) CP Violation and (iii) non-equilibrium. Various different mechanisms are suggested [4,5] and searching for new sources of CP violation is one of the important endeavours. Might there be extra CP violation in the neutrino sector or might some new CP violation show up in precision measurements of particle electric dipole moments, e.g. as complementary probes to high-energy colliders? In dedicated studies of b-quark meson decays at colliders, few standard deviations ‘flavour anomalies’ have been observed in the decays of these mesons to lighter quark mesons and leptons. If confirmed in more accurate data, these might point to violations of lepton universality, viz. the expectation that electroweak gauge bosons have the same coupling to each of electrons, muons and heavier tau leptons. Interestingly, the vacuum of the Standard Model with the particle masses and couplings measured at the Large Hadron Collider, LHC, sits very close to the border of stable and metastable with vacuum stability sensitive to the precise values of Standard Model parameters, especially the Higgs boson and top quark masses [6]. Might this be telling us something about the origin of the Standard Model?

The accelerating expansion of the Universe is believed to be driven by a mysterious dark energy or cosmological constant, the vacuum energy perceived by gravitation, and characterized by a tiny energy scale 0.002 eV. Why is this energy scale so much smaller than the mass scales of particle physics? The particle vacuum contains known potentials associated with QCD and Higgs physics, which involve mass scales about 200 MeV and 246 GeV, respectively, with associated quantum fluctuations. Dark energy [7,8] comprises 68% of the total energy density budget of the Universe today. Might dark energy be time-dependent in the evolution of the Universe and how does it connect to primordial inflation?

Galaxy properties, gravitational lensing and the cosmic microwave background, CMB, all point to the presence of a mysterious dark matter, which is stable on cosmological time scales, electrically neutral and comprises about 80% of the mass budget or 27% of its total energy density budget of the Universe today. Candidates for this dark matter include possible new particles with masses ranging from $10^{-22}\;\mathrm{eV}$ to $10^{15}\;\mathrm{GeV}$ [9,10], primordial black holes [11] as well as models involving possible modifications of General Relativity. The quest to understand this dark matter has inspired a global programme of terrestrial and space-based experiments employing different technologies.

Articles in this volume fall into three main categories spanning the present experimental status and future prospects, as well as key theoretical thinking in these topics.

Jakobs & Zanderighi report on what we know about the Higgs boson, 11 years after its discovery at CERN [12]. Detailed studies of the Higgs and its interactions require precision calculations of QCD and electroweak processes. Besides completing the spectrum of the Standard Model, its coupling to other Standard Model particles (the W and Z bosons, heavy top and bottom quarks, the tau and muon leptons and first measurement of the charm coupling) follow the Standard Model prediction presently to about 10% accuracy. So far, there is no evidence for beyond the Standard Model physics in the presently analysed datasets. The Higgs is the first elementary scalar particle to be discovered. Detailed studies of its properties and interactions including rare decays will continue at the LHC with increased luminosity in the hope of finding possible clues to new interactions. A factor of 20 times more integrated luminosity will be collected than so far recorded, up to a total of about $3000\;{\mathrm{fb}}^{-1}$, by the beginning of the 2040s.

Krizan discusses flavour anomalies in b-quark decays [13]. Curious few standard deviations anomalies between experiment and theory have been observed at BELLE and LHCb which await understanding. These experiments test lepton universality. If the flavour anomalies are confirmed with higher precision, they will challenge our thinking about possible physics beyond the Standard Model.

Ackermann & Helbing [14] concentrate on beyond the Standard Model constraints from experiments such as ICECUBE, the Fermi γ-ray space telescope and KATRIN. Neutrinos and γ-ray signals from space provide complementary inputs with searches for dark matter, magnetic monopoles and Lorentz invariance violation. KATRIN provides the present best constraint on direct neutrino measurements with $m_{\nu }<0.8\;\mathrm{eV}$.

Pokorski discusses the status of beyond the Standard Model theory after discovery of the Higgs boson and taking into account present constraints from collider and precision experiments [15]. Ideas for new particles include possible heavy supersymmetry particles and light mass axions. Vital information will come from future precision measurements of the Higgs including its Yukawa couplings to fermions. How Standard Model like will it remain with increased precision? One finds interesting connections between flavour physics and possible new axion particles.

Bass considers the origin of Standard Model gauge symmetries. Might our gauge symmetries unify in the ultraviolet or might they be emergent below a scale about $10^{16}\;\mathrm{GeV}$? Might the Standard Model work up to these large energy scales? Emergence ideas are connected to the stability of the Higgs vacuum. With an emergent Standard Model the cosmological constant scale 0.002 eV comes out with similar size to the value of tiny Majorana neutrino masses. Ideas how dark matter scenarios might fit with an emergent Standard Model are discussed [16].

Dvali considers the cosmological constant within an S-matrix theory of quantum gravity [17]. In this theory, the cosmological constant will vanish in the ground state. Any cosmological constant observed today would be associated with a coherent state of gravitons with finite quantum break time. Strong CP violation in QCD induced by topological effects would induce a finite cosmological constant. This is taken to cancel through the introduction of light mass axions.

The discovery of gravitational waves [18] has given us a new tool to probe the deep structure of the Universe. Besides black hole and neutron star merger events, gravitational waves also have the potential to teach us about light dark matter, possible (first order) phase transitions in the early Universe and black holes that might have formed before the usually considered stellar origins. An important issue in learning about fundamental physics issues from gravitational waves involves detailed understanding of their waveforms. Heisenberg presents latest the results from General Relativity balance law constraints [19].

The quest to understand dark matter involves collider, underground and astroparticle experiments. Much effort is focused on rare event searches to look for possible dark matter particles in the sub-GeV to TeV mass range with tiny cross sections for interactions with Standard Model particles. These involve new detector developments including Xenon-based and cryogenic detectors.

Baudis discusses the status and prospects for use of Xenon time projection chambers [20] searching for $1\text{--}1000\;{\mathrm{GeV}/c}^{2}$ dark matter candidates, for neutrinoless double beta decay that would give evidence for lepton-number violation possibly hinting towards a Majorana nature of neutrinos, and the detection of neutrinos from the Sun, including the search for non-standard couplings to Standard Model quarks. The focus here is on the PANDAX, Xenon-nT, LZ and DARWIN experiments.

Parallel developments with cryogenic detectors for dark matter searches are discussed by Mokina & Schieck [21]. Here, the CRESST experiment has placed best limits on dark matter masses ranging between 0.25 and $2.5\;{\mathrm{GeV}/c}^{2}$ for proton-only interactions and in case of neutron-only interactions between 0.16 and $1.5\;{\mathrm{GeV}/c}^{2}$. Furthermore, spin-off products based on the CRESST technology are discussed—the NUCLEUS and the COSINUS experiments. The latter, for instance, will check with considerable increase in precision for any seasonal effect in the detector claimed by previous measurements as possible evidence for the Earth passing through possible dark matter in space.

Finally, Malbrunot and collaborators discuss the status and future experimental prospects for investigations of fundamental CP and CPT symmetries in low-energy precision experiments [22]. The electron electric dipole moment measured using molecular ions vanishes to $O\;(10^{-30})\;e\;\mathrm{cm}$, which constrains possible new CP-violating interactions to similar accuracy to the results from LHC high-energy collisions. Spectroscopy of antihydrogen atoms is performed to $O(10^{-12})$ confirming CPT, a fundamental symmetry of relativistic quantum field theories, to this order. Precision experiments with gravitational states of antihydrogen will test the equivalence principle and quantum physics. Progress in these fields requires the development of new detector technologies, with details discussed here.

We hope that bringing these topics together in the same volume will open new synergies between particle and gravity physics, and inspire a new generation of excellent young scientists to join. The idea for the present volume was conceived during discussions that arose at the Humboldt Kolleg meeting *Clues to a Mysterious Universe: Exploring the interface of particle, gravity and quantum physics*, held in Kitzbühel, Austria, in June 2022.

## Data Availability

This article has no additional data.
